# New Genomic Approaches to Enhance Biomass Degradation by the Industrial Fungus *Trichoderma reesei*

**DOI:** 10.1155/2018/1974151

**Published:** 2018-09-24

**Authors:** Renato Graciano de Paula, Amanda Cristina Campos Antoniêto, Liliane Fraga Costa Ribeiro, Cláudia Batista Carraro, Karoline Maria Vieira Nogueira, Douglas Christian Borges Lopes, Alinne Costa Silva, Mariana Taíse Zerbini, Wellington Ramos Pedersoli, Mariana do Nascimento Costa, Roberto Nascimento Silva

**Affiliations:** Molecular Biotechnology Laboratory, Department of Biochemistry and Immunology, Ribeirao Preto Medical School (FMRP), University of Sao Paulo, Ribeirao Preto, SP, Brazil

## Abstract

The filamentous fungi *Trichoderma reesei* is one of the most well-studied cellulolytic microorganisms. It is the most important fungus for the industrial production of enzymes to biomass deconstruction being widely used in the biotechnology industry, mainly in the production of biofuels. Here, we performed an analytic review of the holocellulolytic system presented by *T. reesei* as well as the transcriptional and signaling mechanisms involved with holocellulase expression in this fungus. We also discuss new perspectives about control of secretion and cellulase expression based on RNA-seq and functional characterization data of *T. reesei* growth in different carbon sources, which comprise glucose, cellulose, sophorose, and sugarcane bagasse.

## 1. *Trichoderma reesei*: Environmental and Lignocellulosic Biomass Degrader

*Trichoderma* species are ubiquitous and cosmopolitan. They are very efficient colonizers of a variety of habitats and can be found from the tundra to the tropics [1], especially in lignocellulosic material and plant rhizospheres, and this effectiveness is translated by the ability of competently degrading the available substrate and of secreting different enzymes and metabolites used in the process [2–5]. The capability of growing in such a variety of carbon sources is also due to the high and fast capacity of responding to diverse environmental signals, being able to adapt according to that current background and regulate its growth, conidiation, and the production of enzymes and secondary metabolites. These signals may vary from different nutrients found in the milieu to the absence and presence of light, and adjusting to them is crucial for the survival of the microorganism [2, 4, 6–8].

As a result of this versatility, *Trichoderma* species are very useful in many aspects that range from plant biocontrol [9] to various sorts of industries [10–13], especially for the cellulolytic enzymes produced by them. Among all species from this genus and which are industrially used, *Trichoderma reesei* is the most studied one regarding lignocellulosic biomass degradation, since it is the main producer of cellulolytic and xylanolytic enzymes [14–17]. The ability of growing in a wide range of carbon sources allows great variability in the production of cellulases, since the gene expression and secretion of enzymes are directly dependent on the different chemical signals produced from the diverse substrates. Considering that the plant biomass, one of the most important and complex substrates used by *Trichoderma*, is composed of mono-, di-, and polysaccharides, the different sugars may have different levels of induction or repression of cellulase genes. Some of the cellulase inducers are cellulose, *β*-glucan, xylan, lactose, cellobiose, and sophorose, while glucose is the main repressor carbon source [18]. When *T. reesei* degrades the lignocellulosic biomass, cellobiose may be converted into sophorose by a transglycosylation activity of a *β*-glucosidase [19, 20]. The comparison of the genomes of *Trichoderma* species, including *T. reesei*, suggests they have a mycoparasitic common ancestral, probably from fungi that degrade lignocellulosic material. Considering this, *T. reesei* may have maintained the mycoparasitic characteristic, which allows it to have advantages over other species when competing for substrate, through the conversion of cellobiose into sophorose by transglycosylation to be metabolized [21, 22]. Differently from other fungi, in *T. reesei*, sophorose acts as a very potent cellulase inducer in very low concentrations, being able to induce the expression of some xylanases as well [23, 24].

With the increasing concern about the environmental disadvantages that fossil fuels and nuclear energy represent nowadays, there has been considerable pursuit for new ways of generating large-scale renewable green energy. One of the currently most promising possibilities is the usage of lignocellulosic material from agricultural and industrial waste [25, 26]. This material is mainly composed of cellulose and hemicellulose, which suffer enzymatic hydrolysis and are converted into simple carbohydrate monomers and finally into second-generation bioethanol [13, 27, 28]. However, considering that the plant biomass is highly recalcitrant, large amounts of enzymes are needed during the hydrolysis process, which make the biofuel production economically unfeasible [29–31]. In this context, *Trichoderma reesei* may play an important role in decreasing costs for bioethanol production, whereas it is the filamentous fungus with the greatest capacity of degrading the lignocellulosic biomass [2, 15, 32].

## 2. The Global Analysis of the Enzymatic Repertoire of *Trichoderma reesei*

**Figure 1:**
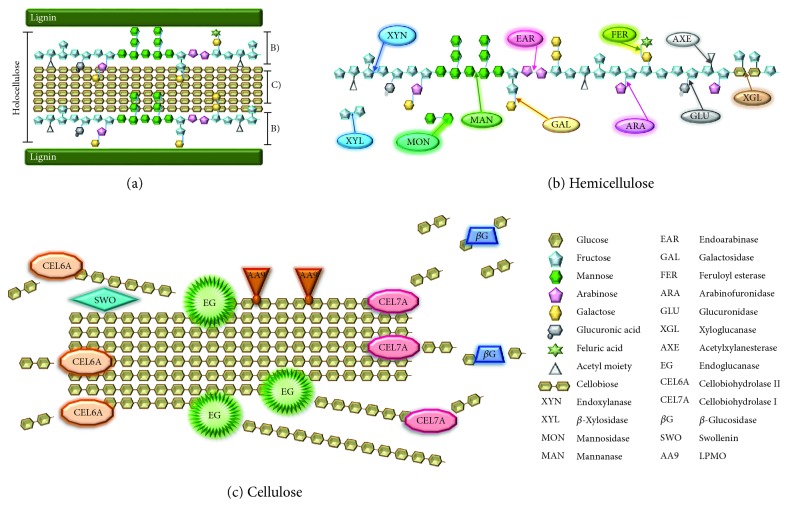
Global regulation of holocellulase expression in *T. reesei*. (a) The schematic structure of the lignocellulosic biomass, which is constituted by lignin and holocellulose, composed of hemicellulose and cellulose. All chains are drawn from the reducing (left) to the nonreducing end (right). (b) Enzymes that attack hemicellulose act in synergy in order to efficiently hydrolyze it and promote a more accessible surface area on cellulose, to enhance cellulase activities. (c) The enzymatic degradation of cellulose: EG acts by cleaving in amorphous regions of the chain, while CEL6A and CEL7A cleave at the nonreducing and reducing ends, respectively. The resulting oligosaccharides from this cleavage are then broken into monosaccharides by *β*-glucosidase, so they become capable of being directly metabolized by the organism. SWO expands the cellulolytic chain to improve cellulase accessibility to it, and AA9 works through bivalent-metallic-ion-dependent oxidative metabolism (based, among others, on [39, 144–146]).

The filamentous fungus *T. reesei* obtains energy through the degradation of the lignocellulosic biomass, composed specially of the cellulose polymer [2, 33, 34]. The cellulolytic enzymes synergistically act to degrade the cellulose polymer. Regarding the site of action, these enzymes are classified in at least three large groups: the endoglucanases, which cleave the internal bonds of the cellulose fiber; the exoglucanases, which act in the external region of the cellulose chain; and *β*-glucosidases, which hydrolyze soluble oligosaccharides into glucose molecules [2, 35] (Figure 1). Recently, LPMOs (lytic polysaccharide monooxygenases) have been suggested to make the cellulose polymer more accessible to the action of traditional cellulases through an oxidative mechanism [36]. In addition to the LPMOs, a protein known as swollenin also participates in the deconstruction of the plant cell wall by breaking the hydrogen bonds between the cellulose microfibrils without hydrolyzing them, thus increasing the biomass degradation efficiency [37] (Figure 1).

In 2012, Häkkinen and coworkers performed a reannotation of all genes encoding enzymes belonging to CAZy (Carbohydrate-Active Enzymes—http://www.cazy.org) in *T. reesei* and identified 201 genes of glycosyl hydrolases, 22 carbohydrate esterases, and 5 genes of polysaccharide lyases [38]. The cellulases produced by *T. reesei* belong to six GH families: endo-*β*-1,4-D-glucanases are found in the GH5, GH7, GH12, and GH45 families, the exoglucanases in the GH6 and GH7 families, and the *β*-glucosidases in the family GH3 [39]. The Cel7a is the dominant enzyme of the cellulolytic complex, comprising about 60% of the total proteins secreted by *T. reesei*, followed by Cel6a (20%), and then the endoglucanases, mostly Cel7b (10%). In fewer amounts, the *β*-glucosidases represent only 1% of the total proteins secreted by this species [40–42].

**Figure 2:**
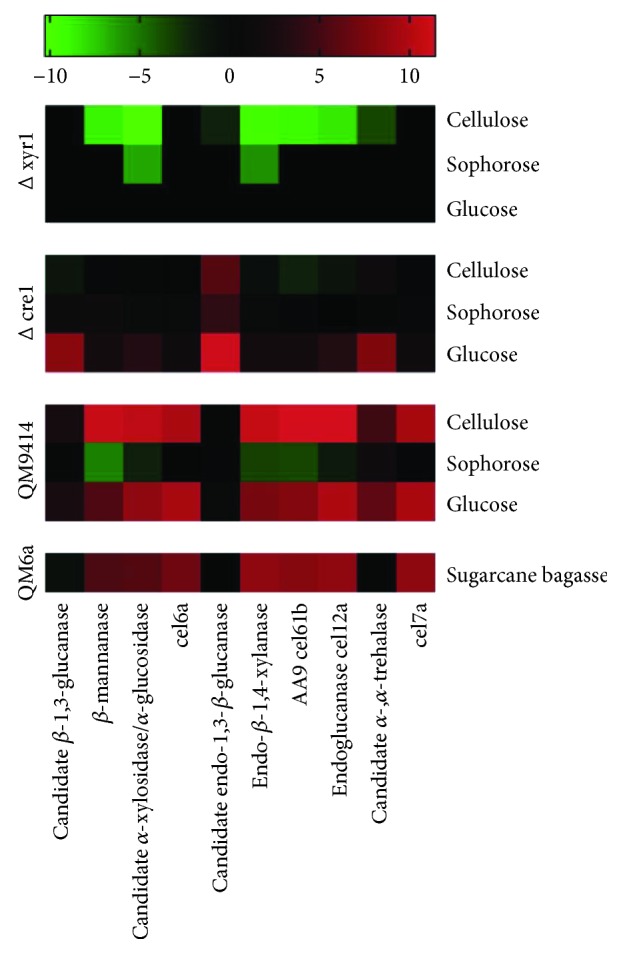
Heatmap expression of Top CAZy-encoding genes differentially expressed in *T. reesei* strains grown in cellulose, sophorose, glucose, and sugarcane bagasse. The results of gene expressions were transformed in Log2FoldChange values and employed to heat map construction using the GraphPad Prism version 7 program (https://www.graphpad.com/).

In 2014, dos Santos Castro and coworkers showed that when *T. reesei* is grown in cellulase inducing carbon sources, such as cellulose and sophorose, CAZyme-encoding genes are highly transcriptionally induced. An opposite profile is observed during growth in glucose, in which most of the genes encoding GH families had low expression. In this study, 61 genes encoding CAZymes were highly expressed in the presence of cellulose in comparison to glucose [21]. Among these, the main gene upregulated was a copper-dependent polysaccharide monooxygenase *cel61b* (ID 120961), almost 3 thousand-fold more expressed in cellulose than in glucose. The endoglucanase *cel12a* (ID 123232) was the second most upregulated gene in this condition, followed by a *β*-mannanase. In sophorose, 58 genes were transcriptionally induced when compared to glucose. The most expressed gene in this condition was the endoglucanase *cel12a* (ID 123232, as well as observed in cellulose). The two cellobiohydrolases of *T. reesei cel7a* (ID 123989) and *cel6a* (ID 72567) ranked the second and third among the most expressed genes in sophorose [21] (Figure 2). In a study by de Paula [43], 98 CAZyme-encoding genes were upregulated during growth in sugarcane bagasse compared to glycerol in the *T. reesei* QM6a strain. The endoglucanase *cel12a* was the third most expressed gene in this condition, similar to that found by dos Santos Castro and coworkers [21] in the presence of cellulose. The two CAZyme genes most expressed in sugarcane bagasse encode a mannanase from the GH76 family (ID 122495) and the hemicellulase *xyn3* (ID 120229). Similar to that found by dos Santos Castro and coworkers in sophorose [21], the cellobiohydrolase *cel7a* was also highly expressed in sugarcane bagasse, achieving 326-fold higher expression in sugarcane bagasse than in glycerol (ID 122495) (Figure 2).

CAZyme gene expression is regulated at the transcriptional level by transcription factors such as XYR1 and CRE1 (for more details, see Transcriptional Regulation of Biomass Degradation). In 2016, dos Santos Castro and coworkers showed that the XYR1 regulator positively regulates expression of at least 61 CAZyme genes in cellulose and 46 genes in sophorose [35]. The genes encoding a candidate *α*-xylosidase/*α*-glucosidase (ID 69944), endo-*β*-1,4-xylanase (ID 120229), and a copper-dependent monooxygenase polysaccharide candidate AA9 (ID 120961) were the major XYR1 target in the presence of cellulose. All of them were more than 500-fold less expressed in the *Δxyr1* mutant compared to the parental QM9414, and the first two genes were also modulated by XYR1 in the presence of sophorose. However, none of these genes are under CRE1-mediated carbon catabolic repression (CCR) [44, 45] (Figure 2). CRE1-mediated CCR was also evaluated regarding the CAZymes of *T. reesei* by Antoniêto and coworkers [44, 45]. In these studies, the most evident CCR occurred during growth of *T. reesei* in glucose [45]. In this condition, several genes encoding CAZymes were repressed by CRE1. A gene encoding a candidate endo-1,3-*β*-glucanase (ID 73256) was the most repressed by CRE1, being more than 2 thousand-fold more expressed in the mutant *Δcre1* compared to the QM9414, followed by another candidate *β*-1,3-glucanase (ID 56418) and a candidate *α*,*α*-trehalase (ID 123456) (Figure 2). The trehalase gene was also under positive regulation of XYR1 [35]. Modulation of the trehalase gene by the XYR1 and CRE1 regulators may be a strategy adopted by *T. reesei* to avoid the unnecessary use of the energy stock when a readily available carbon source is in the culture medium, since this disaccharide acts as an energetic reserve in fungi [44]. Taken together, these results showed that, although the cellulase gene expression profile is similar during *T. reesei* growth in inducing carbon sources such as cellulose, sophorose, and sugarcane bagasse, the mechanisms employed by XYR1 and CRE1 to control the expression of specific genes are dependent on the carbon source available in the environment (Figure 2).

## 3. Involvement of Transporters during the Lignocellulosic Biomass Degradation Process in *Trichoderma reesei*

The process of lignocellulosic biomass utilization involves the capacity of *T. reesei* to sense the insoluble cellulose in the environment and initiates the rapid production of the enzymatic machinery required to breakdown cellulose and offer the nutrients to its growth [46–48]. Appropriated sense of the extracellular insoluble cellulose is key to initiating the rapid synthesis of cellulases by this fungus, and the uptake of soluble sugars released from biomass hydrolysis denote a potential point of control in the induced cascade [48]. In this context, transporter proteins have an important role during the biomass degradation process. Transport systems act in the sensing and uptaking of essential nutrients and ions, allowing excretion of end products of metabolism and toxic substances. Also, these transporters are involved in communication between cells and the environment [49, 50]. It is possible that organisms sense cellulose through recognition of sugars by a transporter in the membrane. In fact, two MFS sugar transporters, *Stp1* and *Crt1*, were implicated in cellulose sensing and cellulase induction in *T. reesei* [48]. Transporter proteins can carry different small molecule inducers from the extracellular environment into the fungus influencing the expression of CAZyme-encoding genes [51–53]. Approximately, 5% (459 genes) of the *T. reesei* genome comprises genes that encode proteins involved in transport [21]. Among these, the largest group of identified transporters belongs to the major facilitator superfamily (MFS), class of sugar transporters, followed by ABC (ATP binding cassette) transporters. These two families of transporter proteins have been most intensively studied among the fungal transporters [34, 54].

The genomes of the filamentous fungus encode large numbers of MFS transporters [55, 56]. These proteins can transport a broad variety of substrates and are divided into 17 distinct families among which three families (1, 5, and 7) are involved in sugar transport into the cell. These transporters carried out the transport of carbon sources, including hexose and pentose sugars, and small soluble molecules in response to ion gradients [50, 57, 58]. In the filamentous fungus, sugar transporters, which belong to the MFS permease family, have a characteristic to recognize and carry more than one type of sugar into the fungal cell. For example, the *T. reesei* STP1 transporter is involved in glucose and cellobiose uptake [48], as well as *Aspergillus nidulans* transporter XtrD was shown to be able to transport xylose, glucose, and several other monosaccharides [59].

Despite advances in studies about the involvement of MFS sugar transporters during biomass degradation, a very few sugar transporters have been functionally characterized in *T. reesei* [48, 60, 61]. A deep transcriptomic and proteomic study investigating the molecular basis for lignocellulose-degrading enzyme production in *T. reesei* during growth in cellulose, sophorose, and glucose revealed new components involved in cellulose degradation, including transporters, while the MFS permeases family was the most present in the different carbon sources evaluated [21]. In this study, MFS permeases differentially expressed in the three carbon sources were analyzed. The gene encoding MFS permease (ID 69957) and MFS permease (ID 76800), targets of light signaling in *Trichoderma*, were highly expressed in cellulose when compared to glucose and sophorose. In sophorose, a gene encoding MFS maltose permease (ID 48444) was highly induced by this carbon source, while the MFS permease ID 76641 was expressed at a higher level in glucose than in sophorose or cellulose [21]. Conversely, cultures with cellulose or sophorose promoted the expression of MFS permeases at similar expression levels, including *crt1* (ID 3405), required for cellulase induction by cellulose and lactose, besides mediating the cellulose sensing process in *T*. *reesei* [48]; *Hxt1*, a glucose permease [60]; *Str1* (ID 50894), a xylose transporter [61]; and MFS ID 79202, which is critical for cellulase production in lactose cultures, although it is related to a sucrose transporter. In addition, the stp1 exhibited a higher level of expression in sophorose than in cellulose, although it is involved in cellobiose and glucose transport [21].

**Figure 3:**
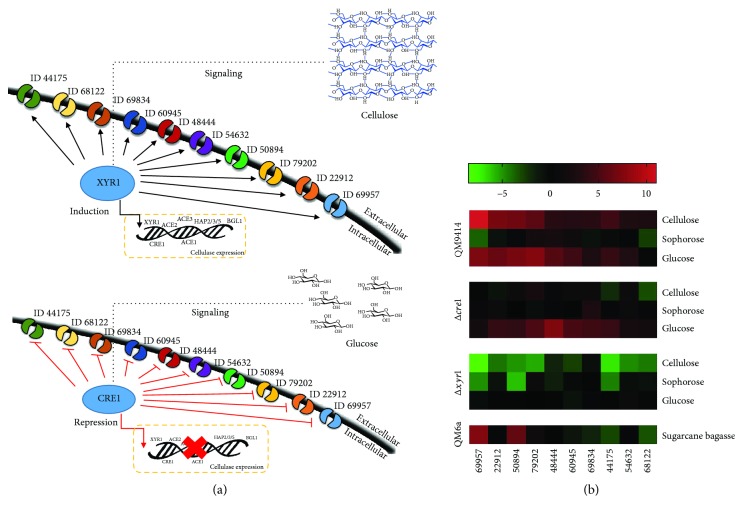
Sugar transporters potentially associated with the degradation of biomass in *T. reesei*. (a) The predicted model of XYR1 and CRE1-mediated MFS permease regulation in *T. reesei* under cellulose inducing condition and glucose repressed condition. (b) Heatmap expression of Top MFS transporter-encoding genes differentially expressed in *T. reesei* strains grown in cellulose, sophorose, glucose, and sugarcane bagasse. The results of gene expressions were transformed in Log2FoldChange values and employed to heat map construction using the GraphPad Prism version 7 program (https://www.graphpad.com/).

In addition, in *T*. *reesei*, the global induction of genes in response to exposure to wheat straw showed that MFS transporters were highly transcribed in straw and repressed in glucose-rich conditions. The genes encoding MFS ID 3405 (*crt1*), ID 50894 (*str1*), and ID 69957 were upregulated in the presence of straw and downregulated in the presence of glucose [62]. Besides, during cultivation of *T. reesei* RUT C-30 strain in sugarcane bagasse, the genes encoding the MFS permeases *ctr1* and *str1* were strongly induced by this carbon source at all the time points analyzed (6, 12, and 24 hours) [63]. The induction of MFS transporters may be controlled by endogenous regulatory systems over a range of in vivo conditions. In *T. reesei*, the modulation of transporter expression was shown to be carbon source-dependent [21, 35, 44]. A global transcriptome analysis of the *Δxyr1 T*. *reesei* mutant strain and the parental strain QM9414 grown in the presence of cellulose, sophorose, and glucose as sole carbon sources showed that genes encoding to proteins belonging to MFS permease superfamily are the most modulated genes by XYR1 (Figure 3). The genes that encode a MFS permease ID 69957, ID 44175, ID 54632, ID 79202, ID 50894, and the ctr1 showed to be strongly repressed by XYR1 in the presence of cellulose [35]. Furthermore, it is possible that the protein transporters are involved in carbon catabolic repression (CCR) in *T. reesei*. During analysis between *Δcre1 T. reesei* mutant strain and parental strain QM9414, grown in cellulose, glucose, and sophorose, the main genes marked by CRE1-mediated CCR encode to proteins belonging to MFS permeases superfamily. Most genes of MFS permeases were upregulated by CRE1 in the presence of glucose. Among these, the expression of the maltose permease gene (ID 48444) and three other MFS permease genes (ID 60945, ID 79202, and ID 44174) was higher in the *Δcre1* mutant than in the parental strain [45] (Figure 3). In sophorose, the gene encoding a xylose transporter gene (ID 104072) was highly repressed by CRE1. During the transcriptional analysis of *T. reesei* grown in wheat straw and glucose, this gene was strongly transcribed in wheat straw and repressed in glucose [62].

Furthermore, a large number of ABC transporters are encoded in *T. reesei* [34]. Generally, ABC proteins are integral to the membrane, acting as ATP-driven transporters for several substrates, including lipids, sugar, drugs, heavy metals, and auxin [64]. In fungi, ABC transporters are involved with the secretion of secondary metabolites, resistance to toxic compounds, and cell signaling [63]. Although the ABC transporters have been shown to be important for different processes in fungi, the role of the transporters belonging to the ABC family is still unclear in *T. reesei*. In this fungus, the ABC transporters showed carbon source-dependent transcriptional regulation, which were upregulated in cellulose and sophorose [35]. In cellulose, the ABC transporter ID 76682 was highly upregulated, while the gene encoding the ABC transporter ID 80028 was upregulated in sophorose [21]. Additionally, in *T. reesei*, it was demonstrated that the transport of molecules by ABC transporters is highly induced by wheat straw and lactose than glucose [53, 65].

The ABC transporters showed to be modulated by transcription factors such as XYR1 and CRE1. In *Δxyr1 T. reesei* mutant strain, the genes encoding ABC transporters ID 55814, ID 60116 (MRP-type ABC transporter), and ID 120114 were downregulated in the presence of cellulose, sophorose, and glucose, respectively. Oppositely, the gene encoding ABC transporter ID 58899 (MDR-type ABC transporter) was upregulated in the presence of cellulose [35]. In *Δcre1* mutant strain, the gene encoding ABC transporter ID 76682 (PDR-type ABC transporter) was upregulated in the presence of cellulose [45] while in sophorose, the major CRE1 repressed gene was ID 76682. However, four other ABC transporter genes (ID 82105, 123293, 73924, and 58899) were repressed by CRE1 in the presence of this carbon source [44]. Among the identified ABC transporters, most of them are correlated with multidrug resistance (MDR), pleiotropic drug resistance (PDR), and multidrug resistance-related protein (MRP) ABC protein subfamilies [66]. Although the ABC transporters contribute to multidrug resistance in microbial pathogens and tumor cells, in fungi, these transporters have been scarcely studied [34]. In other fungi, the ABC transporters are also related with mycoparasitic interaction and antifungal resistance [67, 68], and interestingly, several studies have shown the involvement of ABC transporters with the sugar transport [69]. For example, *Sulfolobus solfataricus* (extreme thermoacidophilic archaeon) uses several sugars as the sole carbon and energy source. This sugar transport is mediated by two families of protein binding dependent ABC transporters that may transport different sugars such as arabinose, cellobiose, maltose, and trehalose [70]. In *Clorynebacterium glutamicum*, Watanabe et al. [71] described a functional characterization of a xyloside ABC transporter and an enhanced uptake of xylooligosaccharides in the presence of a functional xylEFG-encoded xyloside ABC transporter. In *Pyrococcus furiosus*, cellobiose uptake involves an inducible ABC transporter system that not only binds cellobiose but also binds cellotriose, cellotetraose, cellopentaose, laminaribiose, laminaritriose, and sophorose [72].

As exposed here, both MFS transporters and ABC transporters are suggested to be involved during the process of biomass degradation in *T. reesei*. The novel transporters identified offer new perspectives for studies about the involvement of protein transporters in the expression and secretion of cellulase for degradation of lignocellulosic biomass. With this, new proteins might be revealed as involved in the process to sense and transduce signals related to biomass deconstruction, providing future strain improvement for cellulase production. The characterization of these transporters may allow the construction of more efficient strains in the degradation of plant biomass and contribute to the implementation of *T. reesei* in the bioethanol industry.

## 4. Transcriptional Regulation of Biomass Degradation

**Figure 4:**
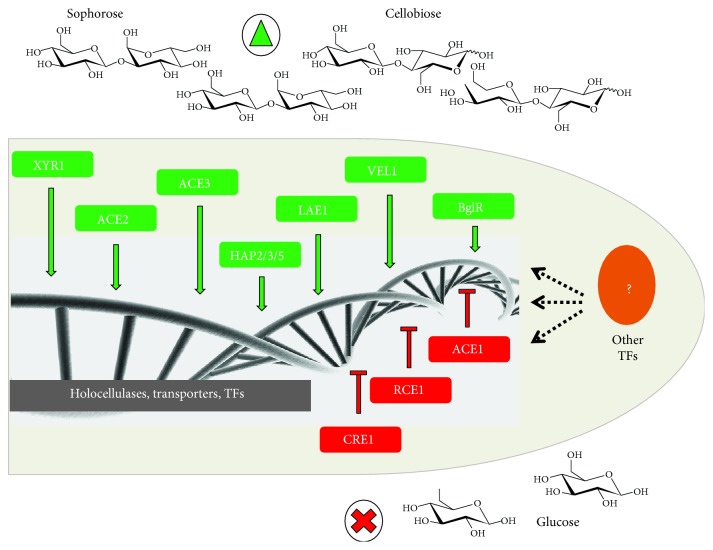
Overview of the transcriptional regulation of biomass degradation. The regulation of cellulose deconstruction involves at least 10 transcription factors: the positive regulators XYR1, ACEII, ACEIII, LAE1, VEL1, BglR, and the HAP2/3/5 complex, as well the repressors ACEI, RCE1, and CRE1. However, several transcription factors still not identified are potentially involved in this mechanism.

The regulation of holocellulose degradation by the fungus *T. reesei* is a highly coordinated phenomenon dependent on the carbon sources available in the medium. In the presence of readily metabolizable carbon sources, such as glucose, the fungus represses the genes accountable for the expression of cellulolytic enzymes as a way of saving energy, while in the presence of inductive carbon sources, such as cellobiose and sophorose, the fungus activates the cellulase production. At the transcriptional level, transcription factors are the key proteins for regulating the expression of genes that act on the hydrolysis of the cellulose polymer [32, 73–75]. In *T. reesei*, ten transcription factors involved in the regulation of this process have been identified so far. They are the positive regulators XYR1, ACEII, ACEIII, LAE1, VEL1, BglR, and the HAP2/3/5 complex, as well the repressors ACEI, RCE1, and CRE1 (Figure 4).

In *T. reesei,* the XYR1 is the master positive regulator of cellulase production, and deletion of this transcription factor totally eliminates the cellulase gene expression and also impairs the induction of hemicellulolytic genes involved with the degradation of xylans and arabinans [73, 76–78]. In 2016, dos Santos Castro and coworkers showed that XYR1 mainly regulates genes belonging to the CAZymes family, in a carbon source-dependent manner [35]. In this work, two xylosidases were the main downregulated genes in the *Δxyr1* mutant in the presence of cellulose (ID 69944; 1260-fold) and sophorose (ID 121127; 209-fold). In addition to CAZymes, transcription factors and transporters were also regulated by XYR1. Besides XYR1, the deletion of the *aceII* transcription factor causes a decrease in the transcription levels of most cellulases and significantly reduces the cellulolytic activity when the fungus is grown in a cellulose-containing medium [79]. Furthermore, the ACEIII transcription factor was more recently discovered, and its deletion was detrimental to the production of cellulases and xylanases in *T. reesei* [80]. It is believed that the HAP2/3/5 complex promotes the formation of an open chromatin structure, necessary for the activation of the transcription process [81]. In addition, the BglR transcription factor acts as a positive regulator of *β*-glycosidases-specific genes [82]. Deletion of the *lae1* reduces the production of cellulases, xylanases, and the auxiliary factors CIP1 and swollenin [83]. Finally, deletion of *vel1* completely decreases the expression of cellulases, xylanases, and *xyr1* genes in the presence of lactose. Interestingly, studies have shown that a combined action occurs between the LAE1/VEL1 complex for regulation of genes involved in biomass degradation [83, 84].

Among the negative regulators of cellulase gene transcription, the main transcription factor is the CRE1 catabolic repressor [75]. In 2014, Antoniêto et al. showed that, in addition to traditional cellulases, CRE1 deletion affects the expression of genes involved in nutrient transport, other transcription factors, and oxidative metabolism. In the presence of cellulose, this regulator represses genes encoding copper transporters and ferric reductase enzymes and, consequently, inhibits the access of the traditional cellulases to the cellulose polymer. In the presence of glucose, CRE1 acts suppressing the expression of genes related to the entry of the inducers into the cell [45]. It was also shown that the transcription factor *xyr1* (ID 122208) is the main target regulator of CRE1 in the presence of glucose, being almost 40 times more expressed in the mutant *Δcre1* when compared to the parental QM9414 [45]. In sophorose, CRE1 mainly modulates CAZYmes and membrane permeases, including maltose permeases that possibly act transporting the disaccharide sophorose [44]. Regarding the other negative regulators of cellulose degradation, studies have shown that ACEI represses the expression of the major cellulolytic genes (*cbh1*, *cbh2*, *egl1*, and *egl2*) and xylanases (*xyn1* and *xyn2*), under the cellulose- and sophorose-inducing conditions [85]. In 2017, Cao and coworkers identified a new transcription factor, Rce1, which acts as a repressor of cellulase gene expression, antagonizing XYR1 by binding to the *cbh1* promoter [86].

Despite the numerous studies about the transcription factors already identified in *T. reesei*, several regulatory proteins involved in the regulation of the lignocellulosic biomass deconstruction have not been characterized yet (Figure 4). In a study of dos Santos Castro and coworkers [21], the expression of 7 genes encoding transcription factors was affected during growth in cellulose. Likewise, 18 genes were also modulated in the presence of sophorose and glucose. In sugarcane bagasse, 88 transcription factors were modulated in the QM6a strain compared to the growth in glycerol [43]. Expression of several genes encoding transcription factors was also under regulation of XYR1 and CRE1. During cultivation in cellulose and glucose, 14 transcription factors were targeted by the CRE1-mediated mechanism of carbon catabolic repression (CCR) [45]. In sophorose, 8 regulators were also regulated by CRE1 [44]. The main positive regulator of cellulase production, XYR1, regulates the expression of other 31 transcription factors in the presence of cellulose, 17 in sophorose, and 7 in glucose [35]. In all these studies, most of the transcription factors modulated have not been characterized yet, which highlights the importance of exploring more about the regulatory proteins involved in the degradation of biomass.

## 5. Epigenetic Regulation of Holocellulase Expression

Another important mechanism of gene transcriptional regulation involves the chromatin modification, a phenomenon that includes the modification of the histones which are the proteins responsible for the DNA packaging. In *T. reesei*, the evidence of nucleosome rearrangement in the promoter region of *cel6a* and *cel7a* suggests that chromatin remodeling is necessary to promote cellulolytic enzyme expression [81, 87]. Interestingly, deleting the methyltransferase LAE1 impairs cellulase expression, while overexpressing this protein increases cellulase expression and changes in the H3K4 methylation pattern in the promoter region of cel5b [83]. This mechanism of regulation was also demonstrated in *M. oryzae*, in which deleting the methyltransferase MoSET1 decreased cellulase induction [88]. Likewise, acetyltransferases are important enzymes involved with chromatin remodeling. In *T. reesei*, acetyltransferases belonging to the GCN5 family are crucial for cellulase expression [89]. During growth of the *T. reesei* QM6a in sugarcane bagasse, de Paula [43] showed that at least 9 genes related to chromatin remodeling were upregulated and 8 downregulated in comparison to glycerol. Of these genes, the acetyltransferase SidF (ID 82628) was almost 40 times more expressed in sugarcane bagasse, reaching the top of the list of genes modulated by this carbon source [43]. Also, dos Santos Castro and coworkers showed that two genes encoding a SWI-SNF chromatin-remodeling complex protein (ID 123327 and ID 122943) were also highly expressed in the presence of cellulose and sophorose [21]. This last gene was a target of CCR by CRE1 under cellulose condition [45]. All these findings reinforce the evidence of the involvement of the chromatin modifications in the mechanism of biomass degradation by the filamentous fungus *T. reesei*.

The genetic engineering has become an important way for improving the production of holocellulolytic enzymes. The modification of gene promoters has also been extensively studied. Recently, Hirasawa and coworkers modified the promoter region of *xyn3* by using the *xyn1* cis-acting region and obtained improved enzyme expression [90]. A study of Zou and coworkers also showed that the CRE1 binding motifs in the promoter region of *cbh1* were replaced by the binding motifs of the positive regulators ACEII and the HAP2/3/5 complex, thus improving the promoter efficiency [91]. In addition, Uzbas and coworkers obtained a *Δxyr1* strain able to secrete the cellulases *cel7a*, *cel7b*, and *cel12a* under control of the promoter regions of two highly expressed genes *tef1* and *cdna1* in a glucose-containing medium [92]. Similarly, the CCR in the presence of glucose was also eliminated by the deletion of CRE1 binding sites and insertion of multiple copies of positive regulator-binding sites in the promoter region of *cbh1*, also increasing the heterologous gene expression in *T. reesei* [93]. These reports highlight the importance of investigating the transcription factors involved in the regulation of biomass degradation and the mechanisms through which these regulators interact with the architectural framework of the DNA in order to improve the holocellulase production.

## 6. Signaling Pathways Involved in Cellulose, Sophorose, Glucose, and Sugarcane Bagasse Recognition

The sensing of the changing environment is an important event for both survival and fungi competition. The fungi have appreciated mechanisms involved in sending signals and reacting to them [34, 94, 95]. Right after the perception of environmental signals, a complex network can be initiated, which is responsible for integrating all signals and promoting a suitable gene response to desirably react to the environment conditions [34]. In *T. reesei*, different signaling pathways have been described to be involved with fungal development and environment sensing. Among these, the signaling pathways dependent on G protein, cAMP, Ras-GTPases, protein kinases, phosphatases, calcium, and MAPK are the mostly characterized intracellular pathways although some aspects remain unclear [95–104]. Several studies showed the involvement of G*α* proteins in the regulation of cellulase gene transcription by light [105, 106]. A functional characterization of a GPCR-encoding gene of *Trichoderma atrovoviride* showed its role in vegetative growth and conidiation [107] and expression of chitinase-encoding genes [108]. Recently, it was demonstrated that two G proteins G*β* and G*γ*s act along with a class I phosducin protein controlling the expression of glycoside hydrolase genes [109]. The cAMP pathway is a highly conserved signaling cascade in which cAMP acts as secondary messenger promoting the integration of different pathways [99]. In *T. reesei,* cAMP levels control cellulase gene expression [110] and according to Nogueira et al. [111], this regulation is in a carbon source-dependent manner.

The cellulase gene expression can also be regulated by the dynamics of protein phosphorylation and dephosphorylation involving protein kinases and phosphatases [34], respectively. Schuster et al. [112] demonstrated the role of protein kinase A (PKA) in the regulation of cellulase expression in the presence of light. Additionally, the deletion of a *T. reesei* casein kinase II promotes the decrease of chitinase expression and repression of sporulation and glucose metabolism [113]. Similarly, He et al. [114] showed that the deletion of protein kinase EKi1 increases the chitinase-encoding gene expression, radial growth, conidiation, and ethanolamine accumulation in the cell wall. The MAPK-mediated phosphorylation can regulate important processes in *T. reesei*. The deletion of the MAPK gene *tmk3* induced a decrease in transcription and cellulase production [115]. Furthermore, it was also demonstrated that *tmk2* is involved in processes regulating cell wall integrity, sporulation and, cellulase production [116]. Recently, it was shown that MAPK IME2 represses the expression of the three main cellulase genes (*cbh1*, *cbh2*, and *egl1*) in *T. reesei* as well as activates the XYR1 and CRE1 expressions [117].

Finally, another interesting signaling cascade involved with the regulation of important processes in fungi is the intracellular calcium-dependent signaling pathway. The calcium homeostasis is essential for organisms, and its intracellular level reflects the environmental changes [118, 119]. In *T. reesei*, calcium-modulated protein (calmodulin) has an important role in xylanase expression [120]. Equally, it was demonstrated that NCS (neuronal calcium sensor-like) interacts with calcineurin and phosphodiesterase, regulating the light-dependent signaling pathway involved with the control of cellulase expression [121]. Recent reports have shown the differential expression of genes encoding calcium transporters and other calcium signaling pathway members during cultivation of *T. reesei* QM9414, *Δxyr1* e *Δcre1* strains in the presence of cellulose, sophorose, and glucose [21, 35, 44, 45]. Similarly, the cultivation of *T. reesei* QM6a strain in sugarcane bagasse, glucose, and glycerol induced the expression of genes encoding Ca^2+^-ATPases and other calcium signaling transporters [43]. These results suggest the involvement of different signaling pathways controlling cellulase expression in the filamentous fungus *T. reesei.*

A deep analysis of gene expression in two functional mutant strains of main transcription factors (XYR1 and CRE1) [35, 44, 45] and two MAPK-encoding genes showed that different signaling pathways and regulatory mechanisms are involved with the regulation of cellulase expression [43]. The main finding of these studies was that the enzyme regulation is carbon source-dependent. The growth of the QM9414 parental strain in the presence of cellulose and sophorose revealed a distinct expression pattern of genes encoding proteins involved with intracellular signaling. Additionally, 50 and 28 genes related to signal transduction were differentially expressed in the presence of cellulose and sophorose, respectively [35]. In cellulose, the main upregulated gene encoded to a conidiospore surface protein cmp1 (ID 72379), which was 21-fold more expressed in the parental strain. Oppositely, the most upregulated gene in sophorose encoded to an unknown protein (ID 73119), which was 8-fold more in the parental strain. Regarding downregulated genes, an unknown protein (ID 65522, 12-fold) was the most repressed in the presence of cellulose. Moreover, the most repressed gene in the presence of sophorose encoded the conidiospore surface protein cmp1 (ID 72379) being 69-fold less expressed in this condition [35]. Curiously, this gene was the most upregulated one when the QM9414 mutant strain was grown in the presence of cellulose. This result suggests that this protein may have an important role in cellulose recognition although the aspects about the regulatory mechanisms involved with the signal transduction remain unclear.

**Figure 5:**
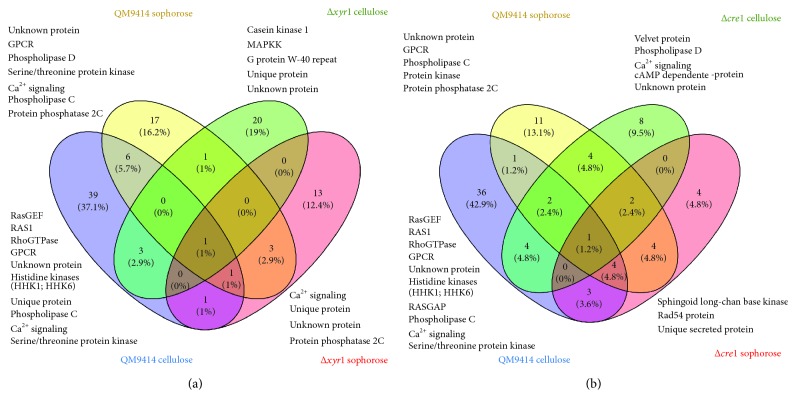
Expression pattern of differentially expressed signaling pathway genes in *T. reesei* during cultivation in cellulose and sophorose. (a) Comparative Venn diagram of expressed genes between the QM9414 and *Δxyr1* mutant strains in the presence of cellulose and sophorose. Venn diagram clustering was designed using Venny 2.1 tools. (b) Comparative Venn diagram of expressed genes between the QM9414 and *Δcre1* mutant strains in the presence of cellulose and sophorose. Venn diagram clustering was designed using Venny 2.1.

Here, we compared the expression patterns of signaling pathway-encoding genes in two functional mutant strains of the XYR1 positive and CRE1 negative regulators of cellulase expression in *T. reesei*. The reported studies have shown there is a specific pattern of signaling pathways for the recognition of different carbon sources (Figure 5). In the QM9414 parental strain, the RAS-GTPAses (RAS-EGF, RAS1, and RhoGTPase) and histidine kinases (HHK1 and HHK6) are the main activated proteins (Figure 5(a)). Moreover, *ras1* gene (ID 67275) and *hhk6* gene (ID 62751) were 8-fold and 2-fold more expressed in the presence of cellulose, respectively [35]. Zhang et al. [97] showed that *ras1* plays important roles in some cellular processes such as polarized apical growth, hyphal branch formation, sporulation, and cAMP level adjustment. Additionally, the deletion of GTPase *ras2* modulates the expression of major cellulase genes and transcription factors in cellulose. The growth and protein secretion of *T. reesei* in cellulose cultures were decreased in *Δrho3* GTPase mutant strain, suggesting *rho3* is involved with secretion processes in this fungus [100].

In the presence of sophorose, we observe distinct expression patterns, being GPCR, phospholipases D and C, and Ca^2+^- signaling the main pathways involved with carbon source recognition (Figure 5(a)). The *Δxyr1* mutant strain demonstrated a distinct pattern of regulation, and, in the presence of cellulose, we observed the activation of casein kinase 1 and MAPKK signaling pathways (Figure 5(a)). The gene encoding to casein kinase 1 (ID 55049) was 2.5-fold more expressed in this condition, and the MAPKK (ID 57513) was increased about 2-fold in cellulose as the carbon source [35]. Wang et al. [113] showed that the casein kinase pathway governs chitinase expression, and beyond that, casein kinase-dependent phosphorylation has been suggested to be an important mechanism of regulation of DNA-binding zinc finger proteins, such as CRE1 [122]. These results suggest that XYR1 is a negative regulator of some genes encoding components of intracellular signaling pathways, such as MAPK and casein kinase cascades, involved with cellulose recognition being this regulation an additional mechanism of transcriptional regulation of cellulase expression.

The functional *Δcre1* mutant strain exhibited a specific expression pattern related to signaling pathways. In the presence of cellulose, the main signaling pathways involved with cellulose sensing were Ca^2+^, phospholipase, cAMP, and velvet-dependent signaling pathways (Figure 5(b)). The Ca^2+^-dependent protein kinase (ID 62181, 5.7-fold) was the most upregulated gene in this condition. Moreover, a cAMP-dependent protein (ID 119614) was 2-fold more expressed in *Δcre1* grown in cellulose [45]. The phospholipase D gene (ID 22331) was 3.8-fold more expressed in *Δcre1* in the presence of cellulose [45]. Finally, the deletion of *cre1* promoted a decrease of 2.3-fold in the expression of regulatory protein velvet 1 (ID 122284). Karimi-Aghcheh et al. [84] showed the deletion of *vel1* completely blocked the expression of xylanases, cellulases, and the regulator XYR1 in the presence of lactose. Since *vel1* was downregulated with *cre1* deletion, this result suggests that CRE1 might indirectly regulate the XYR1 expression in a *vel1*-dependent mechanism, and this may be an unclear mechanism of CCR regulation in *T. reesei*.

**Figure 6:**
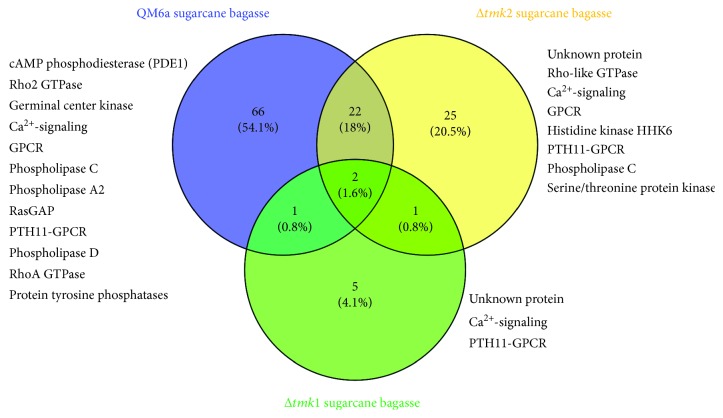
Expression pattern of differentially expressed signaling pathway genes in QM6a parental and two functional MAPK-mutant genes in the presence of sugarcane bagasse. (a) Comparative Venn diagram of expressed genes between the QM6a, *Δtmk1*, and *Δtmk2* mutant strains in the presence of sugarcane bagasse. Venn diagram clustering was designed using Venny 2.1.

The growth of *T. reesei* QM6a in sugarcane bagasse demonstrated a specific expression profile of signaling-encoding genes. In this condition, the expression of genes belonging to cAMP, GTPase, germinal center kinase, calcium, phospholipases, GPCR, protein phosphatases, and PTH11 signaling pathways was observed (Figure 6). Among these, PTH11-encoding genes were an overrepresented category, where eight genes were differentially expressed in this condition. The most upregulated PTH11 (ID 69500) was 14.5-fold more expressed in the presence of sugarcane bagasse [43]. In the filamentous fungus *Neurospora crassa*, PTH11 GPCR seems to be a yet-unclear key role in cellulose recognition and in the control of holocellulytic enzyme expression [123]. De Paula [43] showed in a global transcriptional analysis of two functional mutants for the MAPK signaling pathway that there is a cross talk among different signaling pathways in the presence of sugarcane bagasse. In the *Δtmk2* mutant strain, the main signaling pathways involved with the sugarcane recognition are Rho GTPase, GPCR, histidine kinase, and PTH11 GPCR (Figure 6). Interestingly, PTH11-encoding genes were also overrepresented in the *tmk2* deletion when compared to the parental strain [43]. The deletion of *tmk1* alters the expression of few genes belonging to signaling pathways. Only 4.1% of all genes related with intracellular signaling were involved with sugarcane bagasse recognition in *Δtmk1* mutant strain. Curiously, Vercoe et al. [124] demonstrated in *Ruminococcus flavefaciens*, a cellulolytic bacterium, that the phosphorylation and dephosphorylation dynamics are important for carbon source recognition and regulation of carbon metabolism. Together, our results suggest that in *T. reesei*, there may be a mechanism for carbon source recognition involving PTH11 GPCR, and that this process might be regulated by posttranslational events.

The understanding of how signal transduction pathways modulate cellulase gene expression has acquired attention, such as tools for strain improvement in *T. reesei* [125]. The adequate environmental perception is a crucial step for regulation of gene expression. Thereby, changing transmission of signals aiming at the adjustment of enzyme secretion in response to environmental conditions is an advantageous alternative to enhance the activity efficiency of genes of interest [18]. Different approaches can be employed to improve the production of enzymes by *T. reesei*. One of them is the manipulation of the expression of holocellulolytic-encoding genes by increasing gene expression of activators and/or decreasing expression of repressors that control the expression of the cellulose- and hemicellulose-degrading enzymes. Thus, to carry out this task, a deeper knowledge of holocellulase regulation is crucial [23, 35, 45, 76, 126–128].

In order to accomplish improvement in cellulase expression, different signal transduction mechanisms can be exploited. The holocellulytic gene expression may be regulated by different factors such as light, carbon and nitrogen sources, pH, temperature, inorganic compounds, transcription factors, and epigenetic events [21, 35, 44, 45, 81, 87, 126, 129–133]. These factors and their effects in regulating intracellular signaling pathways have been well studied in *Trichoderma*. As an example, in *T. reesei*, light and photoreceptors BLR1 and BLR2, which belong to light signaling, are known to regulate expression of cellulase genes [134, 135]. Regarding *cbh1* and *cbh2*, the two main cellobiohydrolases, it has been demonstrated that growth of *T. reesei* in the presence of light promotes an increase of 2-fold in gene expression in the presence of cellulose [134, 135]. The signal integration of light and nutrient signaling was an important discovery in the understanding of cellulase gene expression [105, 106]. The G-protein alpha subunits GNA1 and GNA3 were described to be involved in light-dependent regulation of cellulase gene expression. Additionally, transcriptional analysis of the effect of deletion of GNB1 and GNG1 as well as the phosducin-like protein PHLP1 showed that glycosyl hydrolases are the major targets of light-dependent signaling by heterotrimeric G-proteins [109]. The intracellular level of cAMP directly affects cellulase expression [110, 136]. Nogueira et al. [111] showed that intracellular levels of cAMP were 4-fold in the presence of sophorose, and cAMP may regulate secretion of cellulolytic enzymes in *T. reesei* in the presence of this sugar. Interestingly, de Paula [43] showed that deletion of MAPK gene *tmk1* promoted the downregulation of the main holocellulolytic genes of *T. reesei*. These results suggest that TMK1 is a positive regulator of cellulase gene expression and point this gene as a potential target for improvement engineering approaches to enhance cellulase expression in this fungus. Finally, all the knowledge about intracellular signaling pathways and its effects in the cellulase expression in *T. reesei* will provide important insights for metabolic engineering for strain improvement to be used in biotechnological industries.

## 7. The New Players Potentially Involved in *T. reesei* Lignocellulosic Biomass Degradation

In order to survive in different environments, filamentous fungi must be able to sense the surroundings and respond to it accordingly. Once the fungus senses the carbon source (whether it is sugarcane bagasse, cellulose, or sophorose—cellulase inducers), a signaling cascade is activated, and it ends with increasing/decreasing the transcription level of certain genes involved in the degradation of each carbon source. However, all this sensing and responding processes are still not completely characterized. Attempts to solve this puzzle have been made by analyzing transcriptome results, and several studies have shown that *T. reesei* presents genes that code for proteins of unknown function [137].

A differential analysis of expressed genes when *T. reesei* was cultivated in cellulose versus glucose, in sophorose versus cellulose, and in sophorose versus glucose revealed that about 35 to 46% of the genes presented unknown function [21]. Also, when analyzing the top 10 upregulated genes in cellulose, five of them corresponded to protein of unknown function [21]. Among the top 10 upregulated genes in sophorose, there were three of unknown function. These results suggested these genes (were upregulated in inducing conditions) play an important and so far a neglected role in biomass degradation.

**Figure 7:**
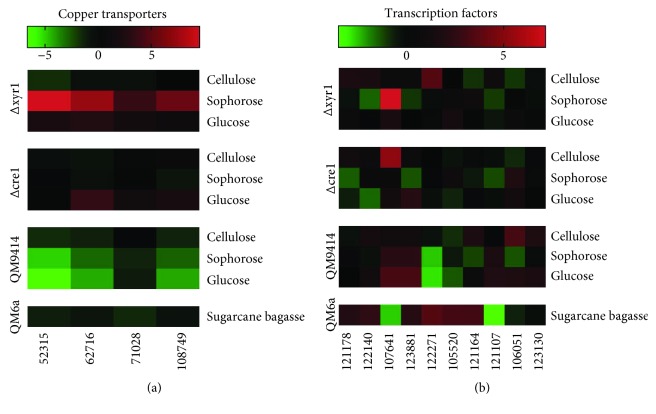
Heatmap expression of new players potentially involved with *T. reesei* biomass degradation. (a) Expression profile of copper transporter-encoding gene differentially expressed in *T. reesei* strains grown in cellulose, sophorose, glucose, and sugarcane bagasse. (b) Expression profile of transcription factor-encoding gene differentially expressed in *T. reesei* strains grown in cellulose, sophorose, glucose, and sugarcane bagasse. The results of gene expressions were transformed in Log2FoldChange values and employed to heat map construction using the GraphPad Prism version 7 program (https://www.graphpad.com/).

Some transporters have been reported to be involved with sensing external carbon sources and thus with the induction of CAZymes [48, 61]. Although the importance of these proteins has been known, specially sugar transporters, for the cellular response to the environment, there are putative transporters that have been differentially expressed [21, 35, 44, 45] and have not yet been characterized.  Figure 7(a) summarizes a few genes that have been expressed in very different levels depending upon the carbon source. Among them, four copper transporters (ID 52315, ID 62716, ID 71029, and ID 108749) had transcriptional levels decreased in the presence of sophorose [21]. Supporting this result, Bak showed in 2015 that a copper transporter was downregulated in *Phanerochaete chrysosporium* cultivated in rice straw compared to no carbon source [138]. In 2016, dos Santos Castro showed that the deletion of *xyr1* caused the transcriptional levels of these genes to be upregulated in the knockout strain compared to the wild type in sophorose [35]. Deletion of the major transcription factor involved in CCR, *cre1*, did not result in changes in these four genes [44, 45]. Therefore, these copper transporters may be involved in biomass degradation since copper is a cofactor required for LPMO activity [139], and the results obtained so far suggest they are regulated (directly or indirectly) by XYR1 but not by CRE1.

Another class of proteins related to biomass degradation regulation and that is still poorly characterized is transcription factor (TF). A lot of effort have been put into discovering new TF that would be important for the regulation of expression of genes that is important for an efficient biomass degradation [73, 80, 82, 140]. Antoniêto et al. [44, 45] and dos Santos Castro et al. [21, 35] studied differential expression in *T. reesei* cultivated in different carbon sources. Also, they have studied two transcription factors, CRE1 and XYR1, and how they affect the transcriptional level of genes related to biomass degradation. Among the transcription factors that showed differences in expression, a few unknown TF presented particularly high differences of expression Figure 7(b). The high expression of these TFs in sugarcane bagasse, cellulose, and sophorose suggests that they can play a role on the regulation of biomass degradation and should be considered targets for further studies [137]. Particularly, gene 107641, classified as a transcription factor by KOG, was repressed in sugarcane bagasse and induced in sophorose. Also, the deletion of *xyr*1 caused this gene (ID 107641) to be highly expressed when *T. reesei* was cultivated in sophorose, a cellulase inductor. Moreover, this TF (ID 107641) was also highly expressed in the knockout of *cre*1 when cultivated in cellulose Figure 7(b). All these results combined suggest that this TF (ID 107641) is involved in the regulation of transcription of enzymes related to biomass degradation and that XYR1 and CRE1 supposedly act as repressors for the expression of this TF. Several studies have identified genes encoding putative fungal C6 zinc finger-type transcription factors enriched among the differential analysis [80, 141–143]. However, characterizing them according to the binding site and which gene expression it regulates is still necessary in order to have a greater knowledge of biomass degradation regulation. Therefore, the identification of novel transcription factors which transcription levels are affected by different carbon sources may be the first step for understanding more deeply how the regulation of transcription occurs. Therefore, these new findings could be used for engineering *T. reesei* in order to have a new hyperproducer strain for the industry to use in biomass degradation with higher efficiency.

## 8. Conclusions

The adoption of the filamentous fungus *T. reesei* as the most important holocellulase producer in biotechnology industries has been stabilized over the years. However, some aspects about holocellulase expression remain unclear. Thus, global transcriptional analyses are an excellent approach to understanding gene expression and promoting the selection of candidate genes for construction of strains producing high levels of holocellulases for plant cell wall degradation. This way, knowledge about the mechanisms involved in the recognition of environmental signals, sugar transport, and transcriptional regulatory events involved with fungal adaptation for different conditions consists in an extremely important step in the fungal biology comprehension. The integration of all these data might contribute to a better understanding of the regulatory mechanisms during lignocellulosic biomass degradation by *T. reesei* facilitating its application in fungal biotechnology.
